# Circadian Synchrony: Sleep, Nutrition, and Physical Activity

**DOI:** 10.3389/fnetp.2021.732243

**Published:** 2021-10-12

**Authors:** Kelly L. Healy, Andrew R. Morris, Andrew C. Liu

**Affiliations:** Department of Physiology and Functional Genomics, University of Florida College of Medicine, Gainesville, FL, United States

**Keywords:** circadian rhythm, synchronization, circadian misalignment, entrainment, asynchrony

## Abstract

The circadian clock in mammals regulates the sleep/wake cycle and many associated behavioral and physiological processes. The cellular clock mechanism involves a transcriptional negative feedback loop that gives rise to circadian rhythms in gene expression with an approximately 24-h periodicity. To maintain system robustness, clocks throughout the body must be synchronized and their functions coordinated. In mammals, the master clock is located in the suprachiasmatic nucleus (SCN) of the hypothalamus. The SCN is entrained to the light/dark cycle through photic signal transduction and subsequent induction of core clock gene expression. The SCN in turn relays the time-of-day information to clocks in peripheral tissues. While the SCN is highly responsive to photic cues, peripheral clocks are more sensitive to non-photic resetting cues such as nutrients, body temperature, and neuroendocrine hormones. For example, feeding/fasting and physical activity can entrain peripheral clocks through signaling pathways and subsequent regulation of core clock genes and proteins. As such, timing of food intake and physical activity matters. In an ideal world, the sleep/wake and feeding/fasting cycles are synchronized to the light/dark cycle. However, asynchronous environmental cues, such as those experienced by shift workers and frequent travelers, often lead to misalignment between the master and peripheral clocks. Emerging evidence suggests that the resulting circadian disruption is associated with various diseases and chronic conditions that cause further circadian desynchrony and accelerate disease progression. In this review, we discuss how sleep, nutrition, and physical activity synchronize circadian clocks and how chronomedicine may offer novel strategies for disease intervention.

## INTRODUCTION

The ability to adapt and respond to changes in environmental conditions is essential for the survival and overall health of all living things. Daily fluctuations in light and temperature provide predictable changes in the environment. Mammals and other organisms have evolved a mechanism by which they coordinate physiological processes, such as sleep and meals, with the light/dark cycle (Takahashi, 2017). The evolutionarily-conserved circadian clock integrates external cues, also known as zeitgebers (time-givers), to coordinate behaviors, metabolism and physiology with the light/dark cycle (Cheng and Cheng, 2021).

While the light/dark cycle has not changed since the circadian clock evolved, some of the zeitgebers that reset our clocks have. Many people stay awake late at night due to social or professional obligations. Shift workers, for example, are consistently awake and often eating or exercising at the wrong time. Light at night and other asynchronous cues misguide our internal clocks (Roenneberg and Merrow, 2016). The resulting circadian desynchrony can have serious consequences, including metabolic disorders, sleep and psychiatric disorders, increased risk of heart attacks and cardiovascular events, and cancer (Scheer et al., 2009; Lee S. et al., 2010). This review focuses on the roles that sleep, nutrition, and physical activity play in synchronizing the mammalian circadian clocks, and how desynchrony can affect human health.

## THE MOLECULAR CLOCK MECHANISM

In nearly every cell in the body, there is an evolutionarily-conserved time-keeping mechanism that is generated by transcriptional activators and repressors. The activators drive expression of their own repressors, creating a self-regulating negative feedback loop. The roughly 24-h oscillating transcriptional activity, regulated by activators and repressors within this transcriptional-translational feedback loop (TTFL), gives rise to circadian rhythms of gene expression (Takahashi, 2017). Clock-controlled genes (CCGs) regulated by the TTFL are largely tissue-specific, and underlie the sleep/wake cycle, feeding and fasting, and other complex physiologies.

**FIGURE 1 |:**
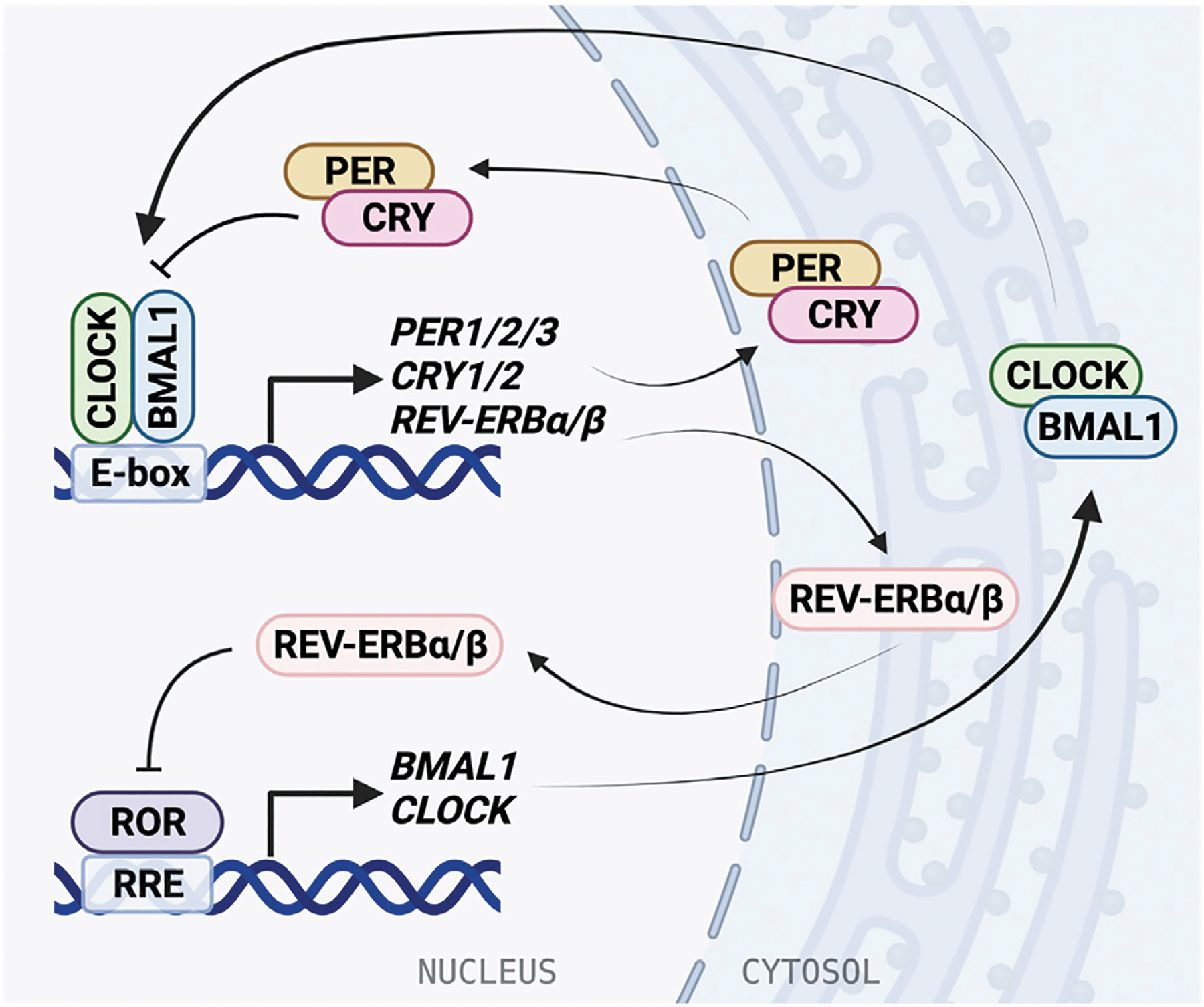
The molecular mechanism of the mammalian circadian clock. The molecular clock is based on a transcriptional/translational negative feedback mechanism and consists of the core loop and two interlocking loops. In the core loop, the BMAL1/CLOCK heterodimer binds to the E-box *cis*-elements and drives transcription of targeted genes, including *PER1/2/3*, *CRY1/2*, and *NR1D1/2 (REV-ERBα/β)*. PER and CRY inhibit BMAL1/CLOCK activity and therefore repress their own expression. In the RRE loop, REV-ERB repressors and ROR activators bind to RRE to regulate *BMAL1* and *CLOCK* expression.

In mammals, the TTFL is formed by core clock proteins that work in pairs to activate and repress transcription (Figure 1). The positive regulatory arm of the TTFL consists of Brain and Muscle Arnt-Like protein 1 (BMAL1), also known as ARNTL, and Circadian Locomotor Output Cycles Kaput (CLOCK). These transcriptional activators heterodimerize in the cytosol before translocating to the nucleus (Lee Y. et al., 2010). Both BMAL1 and CLOCK have a basic helix-loop-helix (bHLH) motif that is responsible for DNA-binding, and two Per-Arnt-Sim (PAS) domains that facilitate protein-protein interactions and heterodimer formation. The bHLH domains fit in the major groove of DNA and recognize the consensus E-box elements in promoter regions to regulate gene expression (Wang et al., 2013). The intrinsically disordered C-termini of BMAL1 and CLOCK allow for promiscuous binding with transcriptional coactivators and repressors to form large complexes (Partch, 2020). Following DNA binding, the transactivation domain (TAD) in the distal C-terminus of BMAL1 initiates transcription (Kiyohara et al., 2006; Park et al., 2015). Coactivators CBP and p300 assist with transcription by stabilizing the BMAL1 TAD (Lee Y. et al., 2010; Dyson and Wright, 2016).

BMAL1/CLOCK activity is negatively regulated by transcriptional repressors PERIOD (PER1/2/3) and CRYPTOCHROME (CRY1/2) (Sato et al., 2006; Takahashi, 2017). BMAL1/CLOCK reciprocates this by binding E-box elements in the *PER* and *CRY* promoters and driving gene expression. Similarly to BMAL1 and CLOCK, PER and CRY also form a heterodimer as they accumulate in the cytosol before translocating to the nucleus. The PER/CRY complex directly binds BMAL1/CLOCK, and is thought to inhibit its transcriptional activity by displacing the BMAL1/CLOCK complex from the DNA (Ye et al., 2014; Cao et al., 2021). CRY acts as the primary BMAL1/CLOCK repressor, binding the CLOCK PAS-B domain and occupying the BMAL1 TAD (Langmesser et al., 2008; Fribourgh et al., 2020), and can repress E-box transcription via a blocking-type mechanism in the absence of PER (Ye et al., 2014; Chiou et al., 2016). Then PER/CRY recruit casein kinases, which phosphorylate BMAL1/CLOCK, disrupting its association with the E-box (Cao et al., 2021). PER acts predominantly as a facilitator for the formation of the BMAL1/CLOCK/CRY complex, while CRY can act independently to inhibit transcription by BMAL1/CLOCK (Xu et al., 2015; Michael et al., 2017). Negative regulation within the TTFL is dependent on interactions between many binding partners and post-translational modifications play an important role in coordinating the timing of association and degradation (Partch, 2020).

An additional oscillating feedback loop exists, in which rhythmic expression of *BMAL1* and *CLOCK* is regulated by two nuclear receptor subfamilies: activators, retinoic acid receptor-related orphan receptors (RORα/β/γ) and repressors, REV-ERBα/β (encoded by *NR1D1*/2 genes). ROR binds the ROR response elements (RRE) in the BMAL1 and CLOCK promoters and is inhibited by REV-ERB (Stratmann et al., 2012; Yoshitane et al., 2019). REV-ERB is regulated in turn by BMAL1/CLOCK through the E-box in its promoter (Ripperger, 2006). The circadian D-box *cis*-element is regulated by activator D-box binding protein (DBP) and repressor NFIL3 (aka E4BP4). Further, circadian expression of *CRY1* is also mediated by the RRE and the D-box, in addition to the E-box (Ukai-Tadenuma et al., 2011; Takahashi, 2017). Rhythmic *DBP* expression is regulated through the *DBP* promoter E-box by BMAL1/CLOCK (Stratmann et al., 2012). These regulatory feedback loops give rise to oscillating expression and activity of clock proteins, allowing for generation of 24-h rhythms.

Posttranslational modifications and timely nuclear localization are key regulatory steps of the clock to introduce delay in transcriptional repression and control of periodicity. Phosphorylation is a common modification of clock proteins. It can cause proteins to be trafficked to, or excluded from, the nucleus, depending on where the phosphorylation occurs (Vielhaber et al., 2000; Akashi et al., 2002; Miyazaki et al., 2004). Phosphorylation can also destabilize proteins, such as in the case of PER1/2 phosphorylation by casein kinase (CK) 1δ/ε (Keesler et al., 2000). CK2 regulates PER2 nuclear accumulation via phosphorylation in the N-terminal (Maier et al., 2009). CK1 proteins also phosphorylate PER and CRY to recruit E3 Ubiquitin ligases. The subsequent degradation of PER and CRY allows BMAL1/CLOCK to resume transcriptional activity (Eide et al., 2005; Takahashi et al., 2008). BMAL1 is targeted by a different ubiquitin ligase, UBE3A, but its degradation also affects clock timing (Gossan et al., 2014). Timely trafficking and degradation of core clock proteins BMAL1, CLOCK, PER, and CRY is what drives generation of 24-h rhythms.

The BMAL1/CLOCK complex transcriptionally regulates thousands of genes, giving rise to a hierarchical network of oscillators at the cellular, tissue, and organismal levels (Zhang et al., 2009, 2014). This network allows for clock regulation of cellular processes, including growth and metabolism, tissue outputs, such as muscle strength and endocrine signaling, and complex physiological outputs like the sleep/wake cycle (Farshadi et al., 2020; Douglas et al., 2021; He et al., 2021). The mammalian circadian clock is able to coordinate these and many other processes with predictable daily changes in the environment. The negative feedback time-keeping mechanism of the TTFL is highly conserved in many organisms and works using the same mechanism in virtually every type of tissue in the body, highlighting its functional significance.

## SYNCHRONOUS CUES

### Entrainment of the Central Suprachiasmatic Nucleus Clock by Light

The circadian system is hierarchical and begins at the suprachiasmatic nucleus (SCN) in the anterior hypothalamus. The SCN is considered the master circadian pacemaker in mammals because it synchronizes all downstream peripheral clocks to the light/dark cycle (Liu et al., 2007b; Mohawk et al., 2012). Direct lesions to the SCN or downstream neurons resulted in the loss of this rhythmic ensemble, which led to the conclusion that the SCN is the master pacemaker (Moore and Eichler, 1972; Stephan and Zucker, 1972). Further, lesions along the light input pathway resulted in free-running rhythms that lacked synchrony to zeitgebers, indicating that, while rhythms can be generated by extra-SCN tissues, the SCN is necessary for entrainment to the light/dark cycle. Although the SCN responds to light, it also can work autonomously in its absence, as demonstrated by mice that maintain locomotor activity rhythms in constant darkness, a free-running condition that reveals the endogenous circadian clock function. These studies, especially those that are more recent and involved genetic perturbation and real-time *in vivo* bioluminescence recordings, led to the understanding that the SCN serves as the central oscillator and systemic circadian coordinator of the organism (Yoo et al., 2004; Sinturel et al., 2021).

Following the discovery of the master clock, SCN transplantation studies established that the donor SCN determines circadian parameters rather than the host. *Tau* mutant hamsters, which have a shortened free-running period, were key to this discovery. SCN transplantation studies involving homozygous *tau* mutant, heterozygous *tau* mutant, and wildtype hamsters with free-running periods of approximately 20, 22, and 24 h, respectively, determined that rhythmicity and period length were reflective of the donor SCN, regardless of the host (Ralph et al., 1990). Transplanted SCN were also shown to restore behavioral rhythms in arrhythmic and mutant mice through neural and diffusible signaling molecules; however, complete restoration of SCN requires intact neural projections, which cannot be maintained through transplantation.

The SCN regulates daily various behavioral and physiological rhythms through a complex neural circuitry within the hypothalamus. Internally, the SCN can be delineated into two subregions composed of various cell types expressing different neuropeptides: the ventral core and the dorsal shell. In the core region, vasoactive intestinal polypeptide (VIP) is the predominant neuropeptide (Yan et al., 2007). Mice deficient of the VIP receptor type 2 (VPAC2) show attenuation of locomotor activity rhythms and significantly impaired ability to phase shift the clock in response to jet lag (Hannibal et al., 2011). These data suggest that VIPergic signaling is crucial for generation of rhythms and phase shift in the SCN. In the SCN shell region, arginine vasopressin (AVP) is primarily expressed. A recent study using conditional *Bmal1* deletion and reporter expression showed that circadian function in AVP neurons, not VIP neurons, is essential for SCN network synchrony and stability of circadian rhythmicity (Shan et al., 2020). The retinohypothalamic tract synapses onto core region neurons, where the light input first resets the ventral (VIP+) SCN and then, through internal coupling, synchronizes the dorsal (AVP+) SCN (Yan and Silver, 2002, 2004). While cell types expressing VIP and VIP receptor type 2 (VPAC2) are unequivocal, in unison they determine periodicity and phase in the SCN (Patton et al., 2020). These regions work together to generate rhythms within the SCN. However, in mediobasal hypothalamus (MBH)-lesioned mice, some locomotor activity rhythmicity and feeding-anticipatory activity was lost, indicating that there are other regions of the brain that play a role in maintenance and generation of circadian rhythms that have yet to be identified (Tahara et al., 2010).

**FIGURE 2 |:**
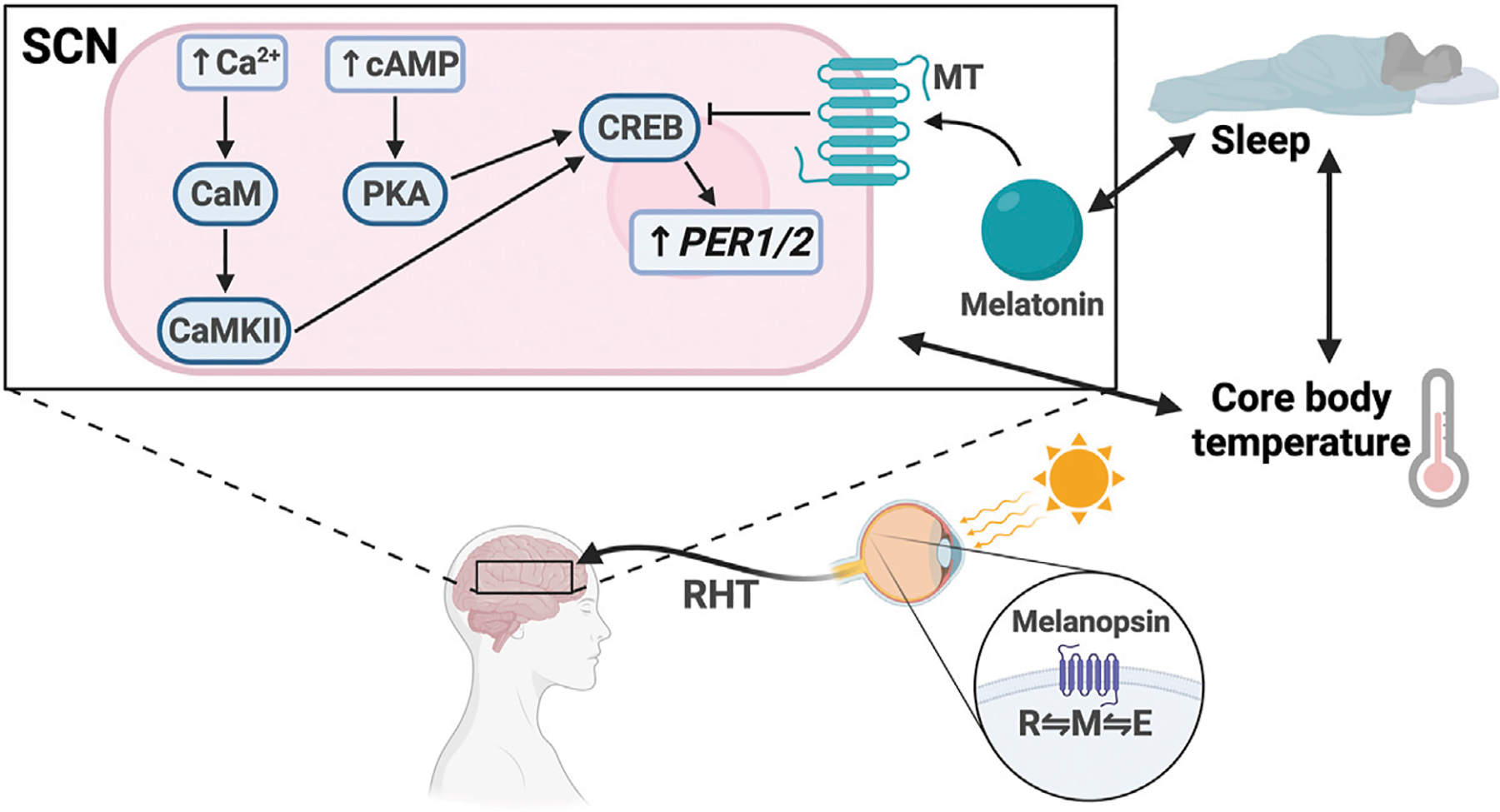
Light entrainment of the SCN and sleep/wake regulation. Light strikes the retina and excites melanopsin, driving it to the M configuration. The signal travels along the retinohypothalamic tract (RHT), resulting in increased intracellular levels of Ca^2+^ and cAMP in the SCN. Ca^2+^ activates calmodulin (CaM) and CaMKII. cAMP activates PKA. CaMKII and PKA activate CREB, which drives *PER1/2* transcription. Melatonin binds the melatonin receptor (MT), which inhibits CREB activation. There is bidirectional regulation between melatonin secretion and sleep, sleep and core body temperature, and core body temperature and SCN signaling.

The light input pathway of the SCN starts at the retina, which is essential for synchronizing the circadian clock to the local light/dark cycle (Liu et al., 2007a) (Figure 2). The retina contains intrinsically photosensitive retinal ganglion cells (ipRGCs), which respond to short-wave light through the G-coupled protein receptor (GPCR), melanopsin (Provencio et al., 1998). ipRGCs can be separated into six classes (M1–6), of which the M1 class contains the highest expression of melanopsin (Lazzerini Ospri et al., 2017). While melanopsin phototransduction appears to differ between classes, the photoisomerization reaction that occurs is the same (Do, 2019). Melanopsin readily interconverts between three states: two resting states, R and E, and an excited state, metamelanopsin (M), that is the intermediate between R and E (Matsuyama et al., 2012). Because short-wave light is more easily absorbed by resting states than the M state, it drives melanopsin to the M state (Matsuyama et al., 2012). When excited, the activated GPCR signals downstream effectors to depolarize cells by opening intracellular ion channels. However, the melanopsin-activated second-messengers have not clearly been identified and evidence suggests that they differ between ipRGC classes (Sonoda et al., 2018).

**FIGURE 3 |:**
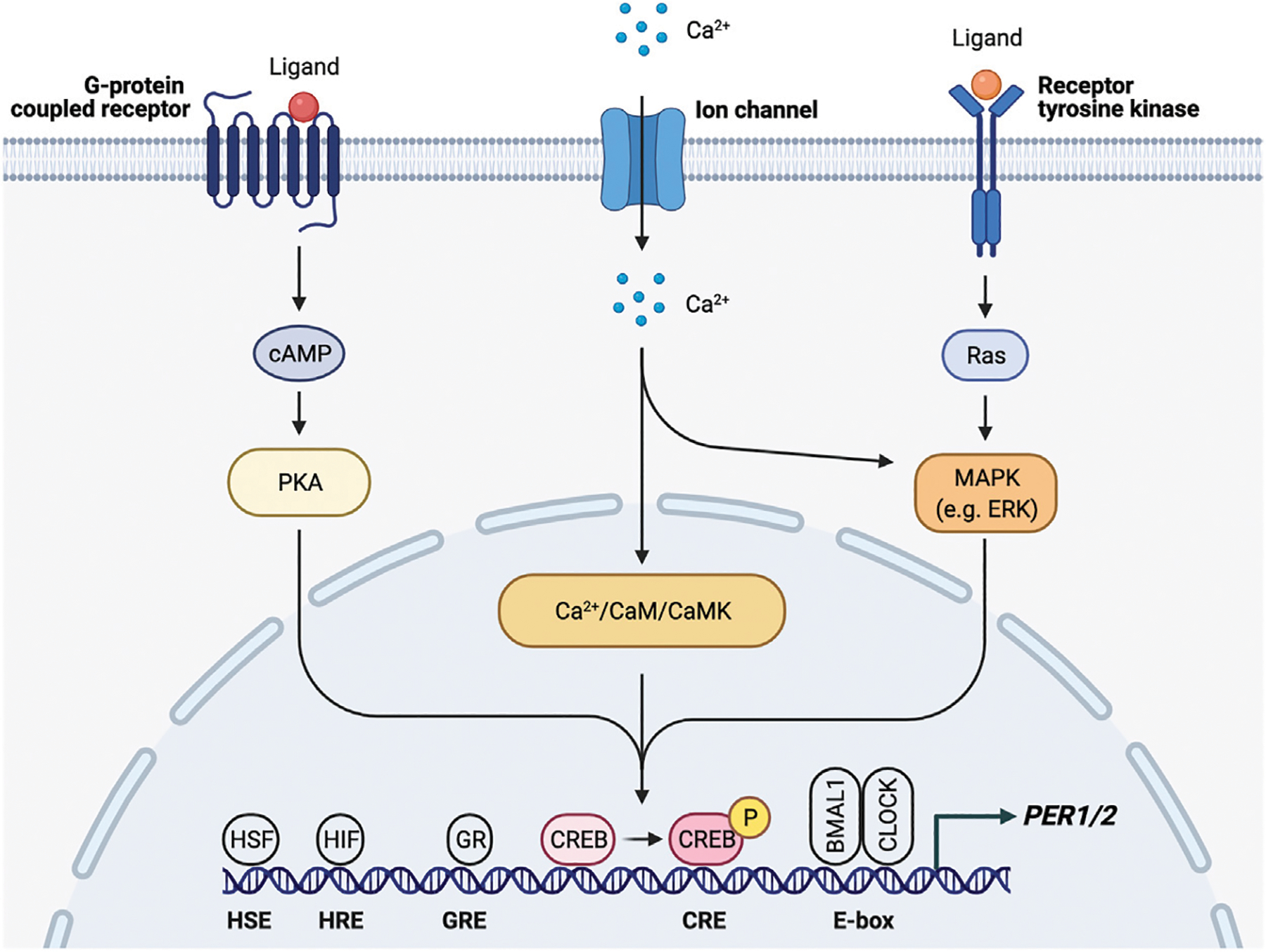
*PER1/2* transcriptional regulation by multiple upstream signals. Ca^2+^/cAMP signaling cascade activates CREB by phosphorylation. Activated CREB binds the cAMP response element (CRE) to drive *PER1/2* transcription. Other proteins (HSF, heat shock factor; HIF, hypoxia inducible factor; GR, glucocorticoid receptor) recognize and bind other consensus sequences (HSE, heat shock element; HRE, hypoxia response element; GRE, glucocorticoid response element).

Regardless of the phototransduction signaling cascade, input signal is passed from the ipRGCs through the retinohypothalamic tract bilaterally to the ventral VIP + SCN, leading to increased *PER1/2* expression via the Ca^2+^/cAMP-CREB pathway (Obrietan et al., 1998; Tischkau et al., 2003; Baek et al., 2018). Increased cellular Ca^2+^ levels activate calmodulin, which subsequently activates calmodulin-dependent kinase II (CaMKII) (Yokota et al., 2001). cAMP response element-binding protein (CREB) is then activated by phosphorylation and binds the cAMP response element in the *PER1/2* promoter, driving *PER1/2* transcription (McNulty et al., 1998; Obrietan et al., 1999; Tischkau et al., 2003) (Figure 3). Increased cellular levels of cAMP also activate CREB in a PKA-dependent manner (Obrietan et al., 1999; Schurov et al., 2002). Rhythmic cAMP-CREB signaling and control of *PER1/2* expression drives SCN synchronization to the light/dark cycle.

It is possible that the SCN could be synchronized by cues other than light, such as meal times or exercise, but light is such a dominant cue that it is difficult to evaluate the strength of other zeitgebers. Despite that, phase shifts in the SCN have been observed in response to a hypocaloric diet under a 12:12 light/dark cycle (Mendoza et al., 2005). This challenges our widely-accepted assumption of light being the single zeitgeber for SCN entrainment. Notably, temperature changes can entrain the SCN *ex vivo*, but only under near-24-h cycles. This is illustrated in SCN explants cultured in 22-–26-h temperature cycles, with temperature drops from 37°C to 31°C (Bordyugov et al., 2015). However, the implications of these temperature entrainment results are unclear since daily changes in core body temperature typically fluctuate ± 0.5° (Barrett et al., 1993). The SCN appears to be impervious to entrainment to physiologically-relevant temperature changes (Buhr et al., 2010). Thus, light remains the most dominant and predictable zeitgeber for the SCN clock.

Circadian rhythms in the SCN clock are robust enough that they can be maintained for weeks or longer *ex vivo* (Evans et al., 2012). This robustness is generated in part by synchronization between SCN neurons (Liu et al., 2007b). Paracrine signaling between SCN neurons drives intercellular coupling (Maywood et al., 2011). Mathematical modeling of coupling between single-cell oscillators demonstrates that coupling strength is proportional to circadian amplitude (Finger et al., 2021). Thus, early efforts in these mathematical models showed that coupling between neurons improves circadian rhythms (Liu et al., 2007b; Mohawk et al., 2012). While coupling was originally thought to be a unique attribute to the SCN, it can also present in peripheral tissues (Sinturel et al., 2021). Recent studies suggest TGF-β signaling as a mechanism for coupling between the SCN and peripheral tissues (Finger et al., 2021). We discuss other major mechanisms of synchronization between the SCN and peripheral clocks in the following sections.

### Synchronization of Peripheral Clocks by the Suprachiasmatic Nucleus

Peripheral clocks, which are found in virtually every tissue in the body, are kept in sync primarily through the SCN. A recent study used *in vivo* bioluminescence imaging to show the SCN is necessary for phase synchrony between organs and lesion of the SCN causes desynchrony of the cellular oscillators, leading to bioluminescence rhythms of reduced amplitude (Sinturel et al., 2021). There appear to be a multitude of pathways that the SCN uses to entrain and synchronize the peripheral oscillators. Concomitantly, there are many response elements in the *PER1* and *PER2* promoters, which provide mechanisms by which intra- and intercellular signals may reset the clock (Figure 3). First, sympathetic nerve activity through catecholamines, such as epinephrine and norepinephrine, is involved in the entrainment of peripheral organs by the SCN by regulating gene expression via a MAP kinase signaling cascade (Terazono et al., 2003). Downstream signals in this pathway lead to elevated levels of *PER2*, thereby resetting the clock. This pathway is such a reliable mechanism of clock resetting that forskolin, a known activator of this MAPK signaling cascade, is often used to synchronize cells in *in vitro* studies (Hu et al., 2021). Resetting through this pathway, however, requires expression of adrenergic receptors in peripheral tissues.

Rhythmic glucocorticoid (GC) secretion is also regulated by the SCN as a means of peripheral clock synchronization. The GC pathway is an ideal candidate for SCN synchronization of peripheral clocks as GC receptors (GR) are found in most peripheral tissues but not the SCN (Cheifetz, 1971; Irvine and Alexander, 1994; Lefcourt et al., 1995; Balsalobre et al., 2000; Cuesta et al., 2015; Boege et al., 2020). GR activation results in transcription of genes that contain glucocorticoid response elements (GREs) in their regulatory regions, including core clock genes *PER1/2* (So et al., 2009) (Figure 3). Through this mechanism, the SCN is able to induce phase shifts in peripheral clocks and synchronize them to the light/dark cycle (Lamia et al., 2011; Cheon et al., 2013). Because the GR provides such a robust mechanism for synchronization, the GC analog dexamethasone, like forskolin, is commonly used to synchronize cells used in *in vitro* studies. However, while GC and adrenergic signaling is important for synchronization, adrenalectomized mice are still able to generate rhythms within peripheral tissues, which indicates that there are other signals and redundant mechanisms that take part in peripheral synchronization (Ikeda et al., 2015).

SCN-regulated daily fluctuations in core body temperature are used to entrain peripheral clocks (Buhr et al., 2010) (Figure 2). Core body temperature shows robust rhythms and typically fluctuates ± 0.5°C each day (Hanneman, 2001; Kräuchi, 2002; Sim et al., 2017). VPAC2-deficient mice showed severe attenuation of core body temperature rhythms, suggesting that the SCN is at least partially responsible for regulating body temperature. This provides a mechanism for synchronization of peripheral clocks through activation of heat shock factor 1, which directly binds to the regulatory sequences within the *PER2* promoter to upregulate *PER2* expression for phase resetting and synchronization (Buhr et al., 2010) (Figure 3).

While the SCN is necessary for phase synchrony between organs, hepatocytes in the liver, for example, can remain phase-coupled even in the absence of the SCN, suggesting there are redundant mechanisms of synchronization for peripheral clocks (Sinturel et al., 2021). Many of these mechanisms are activated by zeitgebers other than the light/dark cycle, and can cause phase shifts in peripheral tissues without signals from the SCN. Below we discuss how sleep, nutrition, and physical activity regulate circadian clocks throughout the body.

### Sleep

Sleep is thought to be regulated by two processes: circadian rhythms, also known as Process C, and homeostatic sleep drive, otherwise known as Process S. This is known as the Two-Process Model, which was first described by Borbely in 1982 (Borbély, 1982). The circadian sleep process is driven by the internal circadian clock and external time of day, whereas the homeostatic sleep process is driven by wake duration. While Process C is mainly controlled by the SCN, Process S is primarily regulated by the ventrolateral preoptic nuclei (VLPO) of the hypothalamus. Sleep debt increases progressively throughout wakefulness and is associated with accumulation of neuromodulatory sleep factors, most notably adenosine (Peng et al., 2020). The mechanisms of these two processes converge through adenosine-mediated regulation of the clock: adenosine binds adenosine A1/A2 receptor, a GPCR expressed in several parts of the brain, and activates Ca^2+^-ERK-AP-1 and cAMP-CREB signaling, the same pathways activated by light (Jagannath et al., 2021). In the SCN, adenosine binds A1 to drive *PER1/2* expression. Adenosine levels, and therefore sleep debt, gradually increase throughout wake time and decrease during sleep. While Process S is cumulative, Process C is time-of-day-dependent and regulated by core body temperature, glucocorticoids, and melatonin.

Melatonin, also known as the “darkness hormone,” is a regulator of the sleep/wake cycle (Figure 2). It is produced by the pineal gland, a neural substrate of the SCN, and is secreted with a circadian rhythm that peaks in the dark phase. Melatonin binds two GPCRs in the SCN: MT_1_ and MT_2_ (Dubocovich and Markowska, 2005). Activated MT_1_ and MT_2_ prevent CREB phosphorylation, thereby downregulating *PER1/2* expression, counteracting the light effect (Kopp et al., 1997; von Gall et al., 1998, 2000; Jin et al., 2003). Melatonin has also been shown to mediate the PI3K/AKT pathway to increase *BMAL1* expression in neuronal cells (Morishita et al., 2016; Beker et al., 2019). It is hypothesized that sleep entrains the SCN through melatonin secretion because MT_1_ and MT_2_ are expressed in the SCN. However, some evidence suggests that melatonin also regulates sleep through Process S by MT_2_-mediated activation of the CaMK/CREB pathway in the VLPO (Wang et al., 2020). More studies are needed to better understand the various roles that MT_1_ and MT_2_ play in different parts of the brain.

Sleep is codependent on SCN-driven thermoregulation, which serves as a zeitgeber for peripheral tissues (Buhr et al., 2010) (Figure 2). Core body temperature (T_b_) decreases as sleep approaches and can influence sleep quality, as demonstrated by the “warm bath effect.” Passive body heating before bed causes a phase delay in T_b_ rhythm and increases slow-wave sleep, also known as deep sleep, a restorative sleep phase which is known to be important for memory (Horne and Reid, 1985; Dorsey et al., 1999; Leong et al., 2019). These temperature changes can entrain peripheral tissues through activation of heat shock factor 1, which induces *PER2* expression by binding to the heat shock element in its promoter (Kornmann et al., 2007; Buhr et al., 2010). Interestingly, a recent study suggests that this temperature synchronization in human-induced pluripotent stem cells occurs through *DBP* rather than *PER2* expression and that HIF1α may be required (Kaneko et al., 2020). Therefore, the physiology of temperature synchronization requires more investigation to fully elucidate the mechanism and identify any differences between different cell types.

In summary, sleep is both an output of and a zeitgeber for the SCN. Sleep further regulates the clock through its impact on the timing of the feeding/fasting cycle and control of energy homeostasis. The sleep/wake rhythm has strong correlations with core body temperature rhythms, but it is unclear when sleep and temperature regulate the clock and when they are regulated by the clock.

### Nutrition

**FIGURE 4 |:**
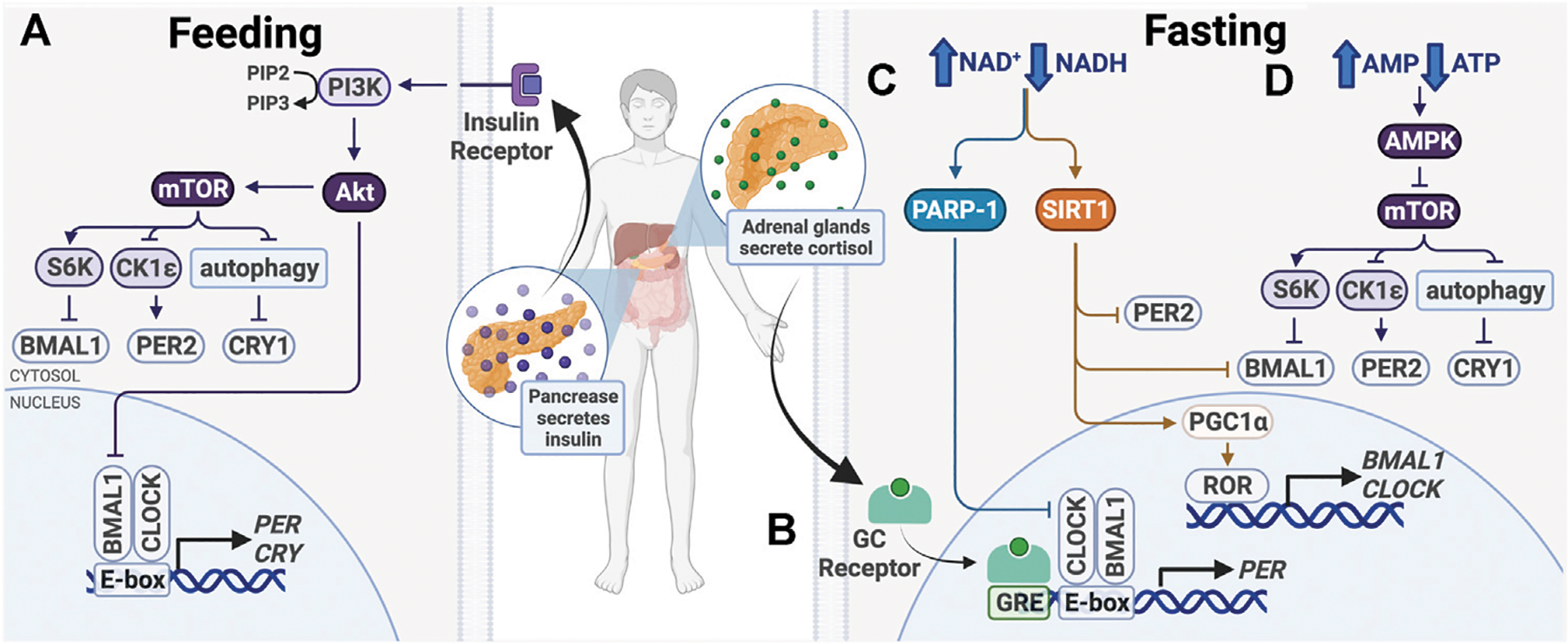
Entrainment of peripheral clocks by feeding/fasting. **(A)** After feeding, insulin activates PI3K/AKT, which inhibits BMAL1 and activates mTOR. **(B)** During fasting, cortisol binds the glucocorticoid (GC) receptor, which binds glucocorticoid response elements (GREs) in promoters to drive *PER1/2* transcription. **(C)** Also during fasting, PARP-1 and SIRT1 are activated in response to an increased ratio of NAD^+^/NADH. Subsequent post-translational modifications alter expression of core clock genes. **(D)** Higher ratio of AMP/ATP activates the AMPK, a mTOR inhibitor.

In recent years, there has been a growing interest in chrononutrition, the study of interplay between nutrition and circadian timing. While meal times do not entrain the SCN, they are a strong zeitgeber for peripheral clocks and become the dominant zeitgeber in the absence of light cues or resetting cues emanating from the SCN, to coordinate circadian timing with feeding and fasting (Hamaguchi et al., 2015; Sinturel et al., 2021) (Figure 4). Elaborate peripheral clock synchronization mechanisms often involve multiple signaling pathways, some of which may be sufficient but not required for phase shifts, and can vary between different tissue types. Recent studies have led to a better understanding at the molecular level of how the nutritional cues affect the circadian clock function. It is likely that there are more synchronization mechanisms that have not yet been identified, but here we discuss what is established so far.

After feeding, a number of signaling pathways take part in nutrient storage and utilization, beginning with insulin release by the pancreas (Figure 4). This signals cells in metabolically active organs, including the liver and skeletal muscle, to take up glucose and upregulate catabolic pathways, such as glycolysis and the pentose phosphate pathway. When nutrients are depleted during times of fasting, glucocorticoid, and glucagon levels increase to initiate anabolic pathways, such as gluconeogenesis. The balance between catabolism and anabolism is critical for the maintenance of homeostasis, which has a strong circadian component. To maintain energy homeostasis, nutrient sensors Sirtuin 1 (SIRT1), poly (ADP-ribose) polymerase-1 (PARP-1), AMP-activated kinase (AMPK), and mechanistic target of rapamycin (mTOR) regulate metabolic pathways. These changes have been shown to modulate circadian clock function and cause clock resetting (Figure 4).

AMPK and mTOR are two master intracellular energy sensors that maintain energy homeostasis and proteostasis. They regulate the clock in response to feeding and fasting through several mechanisms (Figure 4). AMPK is an AMP/ADP-dependent protein kinase that is activated under glucose starvation and operates in a mTOR-dependent manner (Gwinn et al., 2008). AMPK was shown to target CK1ε and CRY1. CK1ε is activated by AMPK-mediated phosphorylation, resulting in enhanced phosphorylation and degradation of PER2. CRY1 is phosphorylated at S71 and S280 by AMPK, leading to its degradation (Um et al., 2007; Lamia et al., 2009). As AMPK activity is rhythmic, it functions in time-of-day-dependent resetting and synchronization of peripheral clocks (Lamia et al., 2009; Um et al., 2011).

mTOR regulates cell growth and proliferation through its activity as a serine/threonine kinase. It phosphorylates transcriptional repressors 4E-binding proteins (4E-BPs) and S6 kinase (S6K), which upregulate protein synthesis. Knock-down of mTOR in osteosarcoma cells, hepatocytes and adipocytes lengthens the cellular circadian period and reduces amplitude, while mTOR activation has the opposite effects (Ramanathan et al., 2018). mTOR affects clock function through posttranslational regulation of BMAL1 and CRY1 (Figure 4). First, S6K was shown to phosphorylate BMAL1 at Ser42, regulating the clock in two ways. BMAL1 phosphorylation destabilizes the protein-DNA interactions and causes its exclusion from the nucleus, thereby downregulating BMAL1/CLOCK transcriptional activity (Dang et al., 2016). Second, S6K was shown to modify BMAL1 in the cytoplasm to promote its involvement in global protein translation, thereby conferring circadian protein synthesis (Lipton et al., 2015). S6K activity on BMAL1 bridges the metabolic mTOR pathway and the circadian clock. Additionally, mTOR-mediated regulation of CRY1 degradation also regulates the clock. mTOR activation and subsequent autophagy inhibition lead to increased CRY1 protein levels, but not mRNA, indicating that that regulation likely takes place post-translationally (Ramanathan et al., 2018; Toledo et al., 2018). Therefore, mTOR and its downstream target take a multifaceted approach to regulating circadian transcription and translation.

In addition to being inhibited by AMPK, mTOR activity is also influenced by insulin and insulin-like growth factor-1 (IGF-1), which is elevated after meals (Levine et al., 2014) (Figure 4). Insulin and IGF-1 activate PI3K-catalyzed phosphorylation of PIP2–PIP3, which binds and activates AKT, an upstream effector and activator in the mTOR pathway (Crosby et al., 2019). Insulin and IGF-1 signaling was shown to upregulate PER2 protein synthesis through mTOR signaling (Crosby et al., 2019). Further, glucagon-CREB/CRTC2 signaling during fasting induces *PER2* expression and PER2 acts to inhibit mTOR activity (Wu et al., 2019). The crosstalk between these signaling pathways provides mechanisms for fasting and feeding to reset the clock and ensure cell and tissue homeostasis.

The pentose phosphate pathway, which breaks down glucose to generate NADH and NADPH, creates another link between metabolism and timekeeping (Figure 4). Enzymes in this metabolic pathway help maintain redox homeostasis within cells. The pentose phosphate pathway regulates the clock through NADPH and the redox-sensitive transcription factor nuclear factor erythroid 2-related factor 2 (NRF2) (Rey et al., 2016). The *NRF2* gene contains an E-box element in the promoter region, and therefore is transcribed by BMAL1/CLOCK (Pekovic-Vaughan et al., 2014; Early et al., 2018). NRF2 in turn downregulates BMAL1/CLOCK by increasing *NR1D1* and *CRY2* expression (Fan et al., 2014; Wible et al., 2018). Inhibition of enzymes in the pentose phosphate pathway or glycolysis decreases cellular levels of NADH and NADPH and abolishes cellular redox rhythmicity (Ch et al., 2021). Furthermore, genetic perturbation of the folate pathway, which is critical for DNA synthesis and protein metabolism, has also been shown to alter the clock (Zhang et al., 2009). The interplay between circadian and metabolic pathways illustrate that the clock is deeply integrated with cell metabolism.

The NAD^+^-dependent protein deacetylase, sirtuin 1 (SIRT1), likewise plays an important role in circadian food-responsive entrainment in peripheral clocks (Figure 4). SIRT1 is upregulated in response to increasing NAD^+^ levels in times of energy stress and nutrient deprivation, and therefore is activated during times of fasting. Overexpression of SIRT1 has been shown to increase physical activity and lower total cholesterol, while SIRT1 deficiency increases blood glucose levels and ROS production, indicating that SIRT1 activation elicits phenotypes beneficial to overall health (Bordone et al., 2007; Wang et al., 2011).

In addition to regulation by cellular NAD^+^/NADH levels, SIRT1 expression and enzymatic activity are also clock-regulated and rhythmic (Nakahata et al., 2008; Zhou et al., 2014). Circadian regulation of SIRT1 in the liver and other peripheral tissues is important for rhythmic control of insulin sensitivity, a major marker for Type 2 Diabetes (Sun et al., 2007; Li et al., 2011; Zhou et al., 2014; Liu et al., 2016). BMAL1/CLOCK regulates *SIRT1* expression by binding E-box elements in the promoter and activating transcription (Zhou et al., 2014; Liu et al., 2016). Thereafter, SIRT1 binds directly to BMAL1/CLOCK to drive expression of nicotinamide phosphoribosyltransferase (NAMPT), an enzyme that catalyzes the rate-limiting step in NAD^+^ recycling (Nakahata et al., 2009). Interestingly, AMPK is necessary for NAMPT activation, suggesting that AMPK also modifies clock timing in part through regulation of cellular NAD^+^ levels (Um et al., 2011). Furthermore, SIRT1 was found to regulate AMPK and mTOR activation, highlighting the bidirectional relationship of many metabolic pathways and their integration with the clock mechanism (Wang et al., 2011; Price et al., 2012).

SIRT1 reciprocates this circadian regulation through its protein deacetylation activity and direct interactions with BMAL1, CLOCK, and PER2 (Asher et al., 2008; Nakahata et al., 2008; Wang et al., 2016) (Figure 4). SIRT1 binds and deacetylates BMAL1 and PER2, with PER2 deacetylation and subsequent degradation being the primary role of SIRT1 in regulating the circadian clock (Asher et al., 2008; Nakahata et al., 2008). This activity is required for oscillations in *BMAL1*, *DBP*, and *PER2* expression (Asher et al., 2008; Nakahata et al., 2008; Foteinou et al., 2018). Nakahata et al. also found that SIRT1 is recruited to the *DBP* promoter E-box in a chromatin complex with BMAL1/CLOCK, indicating that it may regulate BMAL1/CLOCK-driven *DBP* expression through histone deacetylation in the *DBP* promoter (Nakahata et al., 2008). These data suggest that SIRT1 regulates the clock not only at the post-translational level but also through histone modification and epigenetic control.

SIRT1 can also indirectly modify clock gene expression through deacetylation and activation of peroxisome proliferator-activated receptor γ coactivator 1α (PGC-1α) (Nemoto et al., 2005) (Figure 4). The primary function of PGC-1α is to drive mitochondrial biogenesis in response to low cellular energy levels (Than et al., 2011). In myotubes and hepatocytes, PGC-1α activation has also been shown to elevate *BMAL1* and *CLOCK* mRNA and protein levels through activation of RORα/γ (Liu C. et al., 2007). PGC-1α knock-out in the liver and skeletal muscle attenuates rhythmic expression of *CLOCK* and *PER1*, as well as genes, such as pyruvate dehydrogenase kinase 4, that are essential for metabolism (Liu C. et al., 2007). This suggests that PGC-1α is required for circadian regulation of metabolism and SIRT1 appears to be an important regulator in peripheral clocks (Chang and Guarente, 2013).

Similar to SIRT1, poly (ADP-ribose) polymerase-1 (PARP-1) is another NAD^+^ sensor that modulates circadian clock protein activity in response to nutrient depletion. PARP-1 is a versatile enzyme that post-translationally modifies other proteins to facilitate DNA damage repair (Ray Chaudhuri and Nussenzweig, 2017). Its activity in the liver oscillates with a circadian rhythm and can respond to daily changes in cellular NAD^+^ levels. PARP-1 activity peaks at the beginning of the light phase between ZT0 and ZT4 in mice. It binds and poly (ADP-ribosyl)ates CLOCK, disrupting interactions between BMAL1/CLOCK and DNA, subsequently inhibiting BMAL1/CLOCK transcriptional activity and downregulating target gene expression (Asher et al., 2010) (Figure 4).

Hormonal factors such as insulin, IGF-1, glucagon and GCs, and these intracellular nutrient sensors work together to facilitate entrainment of peripheral clocks and align them to the feeding/fasting cycle. Because feeding and fasting are able to entrain peripheral clocks but not the SCN, it is important to be mindful of mealtimes in order to keep peripheral clocks in phase with the SCN (Hamaguchi et al., 2015). Generally, fasting upregulates *PER/CRY* transcription through increased BMAL1/CLOCK activity, but the regulatory mechanisms are complicated and many are intertwined. Moreover, mouse studies have shown that clock changes in response to feeding cues are tissue-specific and can vary drastically (Manella et al., 2021). Clock regulation by metabolic pathways occurs at the transcriptional, translational, and post-translational levels. Likewise, clock regulation also occurs at the intracellular, intercellular, and inter-tissue levels. It is likely that there are more biochemical pathways that alter the clock than what is discussed here, and future studies should explore these mechanisms.

### Physical Activity

**FIGURE 5 |:**
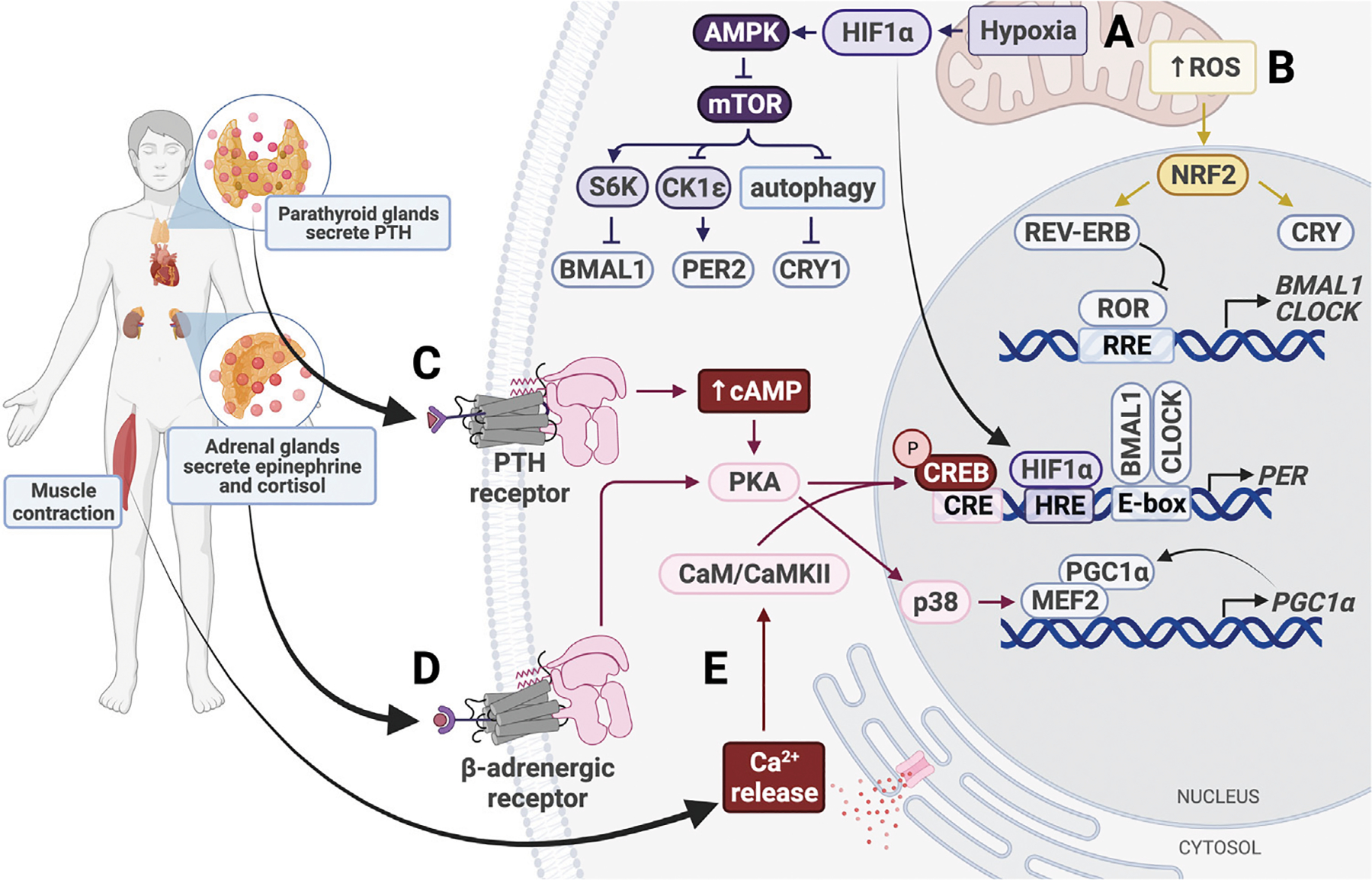
Entrainment of peripheral clocks by physical activity. **(A)** Hypoxic conditions activate HIF1α and AMPK, which inhibits mTOR activity. HIF1α binds the HIF response element (HRE) in *PER1/2* promoters to increase gene expression. **(B)** Increased intracellular levels of reactive oxygen species (ROS) activate NRF2, which subsequently activates CRY and REV-ERB. **(C)** Parathyroid hormone (PTH) binds the PTH receptor to activate the cAMP/Ca^2+^-CREB signaling cascade. Phosphorylated CREB binds the cAMP response element (CRE) in the PER promoter, thereby upregulating PER expression. **(D)** Secreted epinephrine binds the β-adrenergic receptor, which activates protein kinase A (PKA). PKA activates CREB and p38 to alter clock gene expression. p38 triggers activation of the MEF2/PGC1α positive feedback loop. **(E)** Muscle contraction triggers intracellular calcium release, which activates calmodulin (CaM) and calmodulin-dependent kinase II (CaMKII). CaM/CaMKII activate CREB, which upregulates *PER1/2* expression.

Physical activity and other types of physiological stress serve as zeitgebers for peripheral clocks and employ many of the same signaling pathways as fasting (Figure 5). Notably, exercise is able to restore dampened rhythms in peripheral clocks caused by SCN lesion, highlighting its strength in resetting the clock (Sasaki et al., 2016a). Expression of core clock genes *BMAL1*, *CLOCK*, *PER1/2*, and *CRY1/2* is activity-dependent in skeletal muscle and can be modulated through several pathways (Dyar et al., 2015).

Like fasting, physical activity uses GC signaling to synchronize peripheral tissues (Figure 5). In mice, forced exercise has been shown to cause phase shifts in the kidney, liver, and submandibular glands by activating the sympathetic nervous system and the hypothalamus-pituitary-adrenal gland axis (Sasaki et al., 2016a). This exercise-induced phase shift was associated with increases in GCs, epinephrine, and norepinephrine levels, emphasizing the importance of stress response in entrainment of peripheral clocks. In fact, even without exercise, increasing levels of epinephrine alone is sufficient to induce *Per1* expression in the liver and restore damped oscillations of *Bmal1* and *Per2* in SCN-lesioned mice (Terazono et al., 2003).

While the SCN utilizes adrenergic signaling to synchronize peripheral clocks through activation of p38, this pathway can also be activated by exercise and cause phase shifts (Goldsmith and Bell-Pedersen, 2013). Exercise induces secretion of epinephrine and norepinephrine, which activates p38 in a β2AR-dependent manner (Proll et al., 1993; Zheng et al., 2000) (Figure 5). Epinephrine and norepinephrine bind to adrenergic receptors, including the β_2_ adrenergic receptor (β_2_AR; Proll et al., 1993; Zheng et al., 2000). These receptors activate p38, a MAP kinase, in a PKA-dependent manner (Hu et al., 2021). p38 has been shown to activate MEF, which drives transcription of PGC-1α, initiating a positive feedback loop that continues to upregulate PGC-1α expression (Baar et al., 2002; Pilegaard et al., 2003; Akimoto et al., 2005; Miura et al., 2007). PGC-1α is normally used to stimulate mitochondrial biogenesis through CRTC3 in times of energy stress (Than et al., 2011). Upon activation, PGC-1α also elevates *BMAL1* and *CLOCK* expression through RORα/γ, as discussed earlier (Figure 4).

Additionally, the p38 signaling cascade is codependent on nuclear factor of activated T-cells (NFAT) proteins, a family of transcription factors that act as nerve activity sensors in skeletal muscle (McCullagh et al., 2004; Tothova et al., 2006). NFATc1, which is normally phosphorylated and located in the cytosol, is dephosphorylated in response to electrical stimulation and subsequently translocated to the nucleus (Liu et al., 2001; Tothova et al., 2006). Pathway enrichment analysis has shown that NFAT transcription factors upregulate circadian pathways in muscle tissue through CREB and p38 (Dyar et al., 2015). Perturbation of NFAT signaling was shown to affect cell- and tissue-autonomous circadian clock function (Lee et al., 2019). These pathways provide a mechanism by which physical activity resets the clock through GC and epinephrine signaling and electrical stimulation.

Muscle contraction that occurs during exercise resets the circadian clock in muscle tissue in a manner similar to light-resetting in the SCN (Figure 5). Exercise increases intracellular levels of cAMP and stimulates activation of IP3, which binds to calcium channels in the ER to trigger calcium release into the cytosol (Dunbar and Kalinski, 1994; Berdeaux and Stewart, 2012). The calcium-dependent CaMK/CREB pathway is activated in skeletal muscle, resulting in increased *PER2* expression (Small et al., 2020). This pathway is also activated in fibroblasts in response to depleted cAMP following activation of p38 and PKA (Delghandi et al., 2005). In cardiomyocytes, the exercise-induced increase in cytosolic Ca^2+^ levels leads to activation of CAMKII, which then activates MEF2 (Duran et al., 2017; Rodrigues et al., 2018). MEF2 can also be activated by p38, so it is possible that these pathways work synergistically in various tissues to reset peripheral clocks (Yang et al., 1999). While CaMK/CREB signaling has been shown to contribute significantly to clock resetting in skeletal and cardiac muscle, future studies should investigate the role of these pathways in other tissues. For example, exercise-induced parathyroid hormone (PTH) secretion can activate PKA by increasing intracellular cAMP and Ca^2+^ levels through binding of PTH receptor 1, which is highly expressed in the bone and kidney (Abou-Samra et al., 1992; Aghaloo et al., 2006; Tong et al., 2017). Activation of this pathway has been shown to induce Per1/2 transcription activation in chondrocytes, but future studies should determine whether this is sufficient to cause phase shifts in clock gene expression (Hinoi et al., 2006). Additionally, it would be interesting to determine whether the same effect is seen in other tissues, such as kidney and bone.

In addition to inducing epinephrine and GC secretion, exercise also increases cellular energy demand. Lower cellular levels of ATP and NADH activate AMPK and SIRT1 pathways, as discussed (Figure 4). Energy demand is also compensated by upregulation of cellular respiration. Oxidative phosphorylation elevates production of reactive oxygen species (ROS), which cause oxidative stress in the cell and are eliminated following activation of NRF2 (Turrens, 2003; Shadel and Horvath, 2015; Done et al., 2017). NRF2 is a transcription factor that represses inflammation by upregulating expression of genes containing antioxidant response elements. A major transcriptional target of NRF2 is glutathione S-transferase (GSTA4), an enzyme that disposes of ROS by catalyzing their conjugation to reduced glutathione. Besides serving its cytoprotective role, NRF2 also modifies clock gene expression and rhythmicity by upregulating *NR1D1* and *CRY2* expression (Wible et al., 2018) (Figure 5). Interestingly, NRF2 expression and activity are regulated by BMAL1 and CK2, respectively, and antioxidant gene peroxiredoxin-6 expression is upregulated by BMAL1, so the clock plays an important part in maintenance of ROS homeostasis in tissues (Kondratov et al., 2006; Early et al., 2018; Chhunchha et al., 2020; Jang et al., 2020).

Besides causing irreversible damage to DNA, at high levels ROS can reset the clock independently of NRF2. The transcription factor heat shock factor 1 (HSF1) is recruited to BMAL/CLOCK. Hyperphosphorylated HSF1 accumulates in the nucleus and binds promoters of heat-shock genes in a circadian manner (Reinke et al., 2008). The BMAL1/CLOCK/HSF1 complex is subsequently phosphorylated by CK2 at BMAL1-S90 and HSF1-T142 in order to promote transcription of *PER2* (Kornmann et al., 2007; Tamaru et al., 2013) (Figure 3). Redox conditions also alter BMAL1/CLOCK activity through its transcriptional coactivator p300, which acetylates CCG loci and activates BMAL1/CLOCK in a redox-dependent manner (Rey et al., 2016). Therefore, generation of ROS ties exercise and oxidative phosphorylation to the circadian clock.

The increase in oxidative phosphorylation to keep up with the energy demand in response to physical activity also creates hypoxic conditions, which induce expression of hypoxia-inducible factor 1α (HIF1α). HIF1α activates AMPK, an inhibitor of mTOR, thereby slowing cellular growth and proliferation, similar to the response to fasting conditions. Inhibition of mTOR prevents phosphorylation of BMAL1, causing it to remain localized in the nucleus for a longer period of time and ultimately resulting in lengthening of the circadian period and amplitude reduction (Ramanathan et al., 2018). HIF1α also binds directly to E-boxes in a BMAL1-dependent manner to drive transcription of clock genes including *PER2* (Peek et al., 2017). HIF1α is targeted by BMAL1 and, conversely, PER2 is also targeted by HIF1α, which directly binds the HRE-containing *PER2* promoter (Wu et al., 2017) (Figure 3). Stabilizing HIF1α/2α lengthens the period through induction of circadian gene expression, creating a bidirectional relationship between circadian and hypoxic signaling pathways (Peek et al., 2017). Taken together with the p38/NFAT pathways, exercise is able to reset the clock in many peripheral tissues through nutrient starvation/low energy conditions and glucocorticoid signaling.

The implications of clock reset are important to consider when scheduling exercise activities, but when is the best time of day to exercise? There is evidence to support the late afternoon (between 16:00 and 20:00) to be the time for optimal time to maximize performance (Chtourou and Souissi, 2012; Mirizio et al., 2020). In agreement with that, muscle strength appears to peak between 16: 00 and 20:00 (Douglas et al., 2021). However, higher performance does not necessarily correlate with increased output of other benefits of exercise. Therefore, whether the afternoon is the best time of day to exercise is unclear. Regular exercise can improve sleep quality, decrease daytime fatigue, and improve cardiovascular and muscular endurance. Surprisingly, studies show that there is no difference in these improvements between people who exercise in the morning and those who exercise in the evening (Alley et al., 2015; Saidi et al., 2021). Notably, there is contrasting evidence regarding the best exercise schedule for weight loss and lowering blood pressure, which could be due to a number of factors discussed hereafter (Brito et al., 2015, 2019, 2021; Blankenship et al., 2021).

Many variables have brought about controversy in studies investigating the effects of exercise on the clock. First, some authors report that forced exercise causes phase shifts but voluntary exercise does not (Sasaki et al., 2016a). Therefore, the type of exercise must be justified. Second, duration and intensity vary between studies. While exercise duration can be easily controlled for, intensity is more difficult, especially in humans, because physical fitness can vary drastically between individuals (Lewis et al., 2018). Other variables that should be considered in animal studies to ensure reproducibility include room temperature and humidity, number of animals per cage, food availability, and training load progression (Garrigos et al., 2021).

## ASYNCHRONOUS CUES

### Sleep

Because the light/dark cycle is the dominant zeitgeber in SCN entrainment and setting of the sleep/wake cycle, light/dark disruption, as experienced in jet lag and shift work, and artificial light at night (LAN) can disrupt circadian rhythms by altering clock gene expression. A rise in use of electronics has caused an increase in humans’ exposure to LAN, which has been shown to cause phase shifts in the SCN of many mammals, including mice and humans, in a duration- and intensity-dependent manner (Kronauer et al., 2019). Mouse studies have shown that exposure to even dim light during the dark phase can alter Per and Cry expression in the SCN (Shuboni and Yan, 2010). Furthermore, as little as a single day of sleep disruption can alter chromatin accessibility and attenuate rhythmic clock gene expression in the cerebral cortex of mice (Hor et al., 2019). The behavioral phenotype suggested the mice had recovered within 48 h. However, gene expression perturbation persisted for up to 7 days later, demonstrating the long-term effects of acute sleep disruption.

While short-term exposure to LAN is sufficient to cause phase shifts and attenuate rhythms, chronic exposure can increase risk of serious diseases such as cancer, heart attack, and stroke (Brown et al., 2009; Lee S. et al., 2010; Arendt, 2010). This is especially problematic for night shift workers and those who experience chronic jet lag, such as flight attendants. Studies have shown that melatonin suppression, which occurs in response to LAN, promotes carcinogenesis, while melatonin treatment can attenuate tumor growth (Davis and Mirick, 2006). Shift work is classified as a carcinogen, and the risk of cancer, as well as heart disease, further increases with the time spent carrying out shift work (Schernhammer et al., 2001; Megdal et al., 2005; Davis and Mirick, 2006; Vetter et al., 2016; Yousef et al., 2020). When sleeping out of phase, many clock genes lose rhythmicity (Archer et al., 2014). This loss of clock gene expression is associated with poorer cancer prognosis, likely due to loss of *PER2* rhythmicity specifically (Cadenas et al., 2014). PER2 is thought to act as a tumor suppressor, in part by stabilizing p53, but also through regulation of the cell cycle (Wood et al., 2008; Xiang et al., 2008; Gotoh et al., 2014; Farshadi et al., 2020). Downregulation of *PER2* increases Cyclin D and Cyclin E levels and increases the rate of tumor growth in breast cancer, disrupts DNA damage repair pathways and results in increased tumor growth (Fu et al., 2002; Xiang et al., 2008; Yang et al., 2009). It is also possible that internal circadian misalignment causes cancer via genomic perturbation. This is supported by the fact that genomes of night shift workers are significantly hypomethylated compared to those of day shift workers (Bhatti et al., 2015). Therefore, shift work, light at night, and chronic jet lag may increase the risk of cancer and other diseases through several mechanisms, including rhythmic expression attenuation, downregulation of clock genes, and covalent modifications to DNA. Given that shift work comprises many essential jobs that cannot end with the typical workday, solutions should be identified to mitigate the effects and reduce the risks of chronic jet lag. Additionally, the association between shift work and cancer requires further investigation because there is also evidence that shift work does not significantly contribute to carcinogenesis (Yang et al., 2021).

In addition to environmental factors, heritable mutations can also disrupt sleep. This is illustrated by familial advanced sleep phase syndrome (FASPS), which is characterized by a significantly advanced shift in the daily sleep/wake cycle (Jones et al., 1999). Sleep duration is normal in FASPS, however, a *PER2* mutation leading to decreased phosphorylation by CKIε causes a phase advance (Toh et al., 2001). Conversely, in familial delayed sleep phase syndrome (DSPS, a CKIε substitution mutation at Ser408 can increase the phosphorylation rate by 1.8-fold compared to wild type and delay the phase of sleep onset/offset (Takano et al., 2004). DSPS can also be attributed to a single point mutation that disrupts a splice site in *CRY1*. This leads to deletion of exon 11 (CRY1Δ11) and gives rise to a significantly longer circadian period (Patke et al., 2017). In addition to causing insomnia, CRY1Δ11 is also associated with attention-deficit/hyperactivity disorder (ADHD), major depressive disorder, and anxiety (Onat et al., 2020). Because DSPS is characterized by the resulting phenotype and not the underlying genetic perturbation, it can also be caused by a variation in the *PER3* gene. The frequency of a shorter polymorphic repeat region in *PER3* was found to be significantly higher in a population of DSPS subject group compared to controls (Archer et al., 2003). However, this association weakens with age (Jones et al., 2007). Despite the potential underlying differences behind DSPS, the preference for later wake time can affect quality of life, so it may be useful identifying genetic markers that predispose individuals to DSPS.

Mental health disorders can also have a significant impact on sleep homeostasis, and therefore clock function (Liberman et al., 2018). For example, sleep disruption is an early symptom of psychosis (Lynch et al., 2018). This may be due in part to N-methyl-D-aspartate receptor (NMDAR) dysfunction, which is associated with psychiatric disorders and suspected to impact sleep regulation (Wang et al., 2020). NMDA increases intracellular Ca^2+^ levels in the SCN, resetting the clock through activation of the Ca^2+^/cAMP-CREB signaling pathway (Schurov et al., 2002) (Figure 2). NMDAR antagonism has been shown to lengthen the circadian period, but that effect is reversed with melatonin treatment (Wang et al., 2020). Attention deficit hyperactivity disorder (ADHD) is also strongly associated with disruption of sleep and circadian rhythms, specifically *BMAL1* and *PER2* rhythms (Baird et al., 2012). Surprisingly however, rather than reversing these phenotypes, stimulants used to treat ADHD can worsen sleep disturbance and cause further phase shifts (Coogan et al., 2019). ADHD is thought to be caused by a reduction in dopaminergic signaling (Volkow et al., 2009). Likewise, depression, which is also associated with insomnia, has been attributed to dysregulation of dopaminergic signaling (Grace, 2016; Li et al., 2016). Therefore, ADHD, depression, and other mental health disorders may cause circadian disruptions due to decreased activation of the Ca^2+^/cAMP-CREB pathway, as dopamine has been shown to activate this signaling cascade (McNulty et al., 1998; Schurov et al., 2002). More research is needed to fully understand the interplay between psychological disorders and the sleep/wake cycle.

Unfortunately, aging is also associated with lower sleep quality. Specifically, sleep fragmentation and also impaired cognitive function have been attributed to aging (Yoon et al., 2003; Kaneshwaran et al., 2019). This sleep fragmentation is thought to alter circadian rhythms via Ca^2+^/cAMP-CREB signaling, as prevention of CREB phosphorylation by melatonin was shown to be attenuated in the SCN of aged mice (von Gall and Weaver, 2008). Nevertheless, more research is needed to fully understand sleep dysregulation and attenuation of circadian rhythms with aging. Notably, there is a bidirectional relationship between aging and sleep disruption. Chronic sleep deprivation has proven to be detrimental to long-term memory and to impair overall cognitive function, eventually leading to dementia (Lo et al., 2016; Cousins and Fernández, 2019; Zhang et al., 2019; Garcia-Aviles et al., 2021).

Bettering our understanding of how aging and other disease states affect the clock will be useful in identifying interventions that will lessen the detrimental effects on sleep and clock function. There are many factors, environmental or otherwise, that affect both sleep quality and quantity. However, it is important to minimize sleep disruption whenever possible to enhance circadian clock function and improve overall health.

### Nutrition

Under normal conditions, the feeding/fasting cycle is aligned with the sleep/wake and light/dark cycles. However, mistimed meals can cause phase shifts in peripheral tissues that can have serious consequences on overall health. Asynchrony between the SCN and peripheral tissues, otherwise known as internal circadian misalignment, has been shown to lower glucose tolerance, cause a phase shift in cortisol secretion, increase the risk of obesity, and increase the overall levels of triglycerides (Marcheva et al., 2010; Morris et al., 2016; Grant et al., 2021). These physiological changes consequently increase the risk of obesity, diabetes, cardiovascular diseases, cancer, and other age-linked diseases (Davis and Mirick, 2006; Scheer et al., 2009; Haus and Smolensky, 2013; Shi et al., 2013; Gan et al., 2015).

Meals are a dominant zeitgeber for peripheral clocks, and mistimed meals can cause drastic phase shifts of up to 12 h or more (Damiola et al., 2000; Le Minh et al., 2001; Sujino et al., 2012; Wolff and Esser, 2012; Salgado-Delgado et al., 2013; Wehrens et al., 2017). Peripheral clocks are sensitive enough to changes that skipping breakfast for even 1 day can significantly alter clock gene expression (Jakubowicz et al., 2017). This is especially relevant to shift workers who are more likely to regularly have mistimed meals. The negative effects of restricted feeding (RF) during the sleep phase, also known as the inactive phase, have been well documented in rodent models. Disrupting the clock through RF can lower insulin sensitivity, reverse core body temperature cycling, and increase total caloric intake (Satoh et al., 2006; Bray et al., 2013; Zhou et al., 2014; Liu et al., 2016). In humans, eating at night has been shown to attenuate rhythms and decrease total sleep time (Morris et al., 2016; Grant et al., 2021). Even delaying meals by just a few hours can cause phase shifts in peripheral tissues (Wehrens et al., 2017).

Intermittent fasting (IF) is a habit that restricts meals to roughly 6–12 h per day. It has been found to provide numerous benefits in weight loss and anti-aging efforts, including increased insulin sensitivity, thereby reducing the risk of diabetes (Schenk et al., 2011). Further, in mice, time-restricted feeding was shown to lower fat accumulation, improve glucose tolerance, increase insulin sensitivity, and reduce inflammation (Chaix et al., 2014, 2019). However, in human studies, many participants restrict meals to the afternoons and evenings, delaying time cues that would normally reset peripheral clocks in the morning. Rather than improving health, eating later dinners can increase the risk of obesity (Tahara and Shibata, 2013). Shifting the time of restricted feeding to start at the beginning of the active phase could allow participants to continue reaping the benefits of fasting without disrupting the circadian clock (Regmi et al., 2021). A recent clinical trial showed that early time-restricted feeding, a form of IF, improved insulin sensitivity, blood pressure, and oxidative stress levels without causing weight loss (Sutton et al., 2018). However, a study involving 24-h fasts after breakfast resulted in decreased expression of antioxidant enzymes, indicating that there can be adverse effects to IF, possibly due to fasting for too long (Liu et al., 2021). Finally, there is contradictory evidence regarding the effects of weight loss in response to time-restricted feeding in humans (Adafer et al., 2020). This may be due, at least in part, to the differences in populations that were evaluated, the length of time throughout the day when eating was permitted, and the duration of the studies. Therefore, more research is needed to optimize the timing and nutritional value of diets that provide the best outcomes in disease prevention and anti-aging. There are also many factors that prevent participation in IF, including cost, perceived health benefits, and wake/sleep time, that must be addressed before IF can be implemented on a larger scale (Jefcoate et al., 2021; Veronda and Irish, 2021).

In addition to timing, it’s also important to be mindful of the nutritional value of meals. Increasingly popular diets often require participants to severely restrict particular food groups. High-fat diets, such as the ketogenic diet, have proven to have many benefits. For example, the ketogenic diet was effective in treating epilepsy and Type 2 Diabetes in young and middle-aged adults (Napoleão et al., 2021). While ketosis-inducing diets can improve metabolic and other syndromes, they can have negative effects on the circadian clock. For example, a high-fat diet has been shown to lengthen the free-running period in mice as well as attenuate rhythms of plasma insulin, in part through inhibition of AMPK and pentose phosphate pathway enzymes (Kohsaka et al., 2007; Stucchi et al., 2012). Further, obesity induced by a long-term high-fat diet was shown to increase the expression levels of core clock genes *Bmal1*, *Dbp*, and *CK1ε* in mouse livers and *Bmal1, Clock, Per1/2/3, Cry1/2, Dbp*, and *CK1ε* in the kidneys (Hsieh et al., 2010). On the other hand, a balanced diet containing carbs, proteins, lipids, and vitamins/minerals was shown to be important for liver entrainment and sufficient to cause phase shifts (Hirao et al., 2009). Furthermore, caloric restriction was shown to activate SIRT1, which is known to have many benefits, including lower cholesterol, lower plasma glucose levels, and increased insulin sensitivity (Bordone et al., 2007; Schenk et al., 2011). Therefore, it is important to balance nutritional intake with physiological requirements.

Scheduling meals and drug administration for appropriate times can help peripheral clocks stay synchronized to the SCN. Preventing circadian misalignment is important for maintaining overall health and maximizing physiological outputs such as sleep and muscle strength (Leatherwood and Dragoo, 2013; Morris et al., 2016; Song et al., 2017; Aoyama et al., 2021). Circadian misalignment has also been shown to accelerate the progression of various diseases and therefore should be minimized whenever possible (Tahara and Shibata, 2016). Because of the crosstalk between metabolic pathways and the circadian clock, chrononutrition shows high potential for prevention and treatment of disease states (Barrea et al., 2021).

### Physical Activity

Timing of physical activity is important in regulating the circadian clock, as the direction in which exercise-induced phase shift occurs is time-of-day-dependent. Exercise sessions in the early evening (before melatonin onset) have been shown to cause significant phase advance, while bed rest and exercise late at night can cause phase delays in healthy adult men (Buxton et al., 2003). Timing of exercise has also been shown to affect which metabolic pathways are activated in response to activity. For example, forced acute exercise in the early rest phase was shown to stimulate carbohydrate metabolism in the skeletal muscle of mice. In contrast, forced exercise in the early active phase stimulated metabolism of lipids, amino acids, and ketones, as well as activated the HIF-1a signaling cascade (Sato et al., 2019). However, while exercise can be a strong zeitgeber for some peripheral clocks, it does not appear to be as effective for entrainment as meal times. In fact, exercise-induced phase shifts in peripheral tissue can be dependent on meal times (Sasaki et al., 2016b). Because physical activity is a less dominant zeitgeber than feeding, scheduled exercise cannot effectively entrain peripheral clocks if it is not in phase with meal times. In summary, physical activity can either couple peripheral clocks to the SCN or cause desynchronization. This affects not only shift workers, but also anyone who exercises late at night.

## CHRONOPHARMACOLOGY AND CHRONOTHERAPEUTICS

In addition to feeding and exercise, some drugs that act on pathways modulating circadian timing cause phase shifts or other clock dysfunction. An example is PARP-1 inhibitors, a class of anti-tumor drugs that sensitize cancer cells to DNA damage caused by chemotherapy or radiation (Malyuchenko et al., 2015). Because PARP-1 acts primarily to detect single-and double-strand DNA break, inhibiting its activity can result in failure to repair damaged DNA in cancerous cells, inducing cell death. First-generation PARP-1 inhibitors are analogs of nicotinamide, one of the products of the reaction catalyzed by PARP-1. Nicotinamide, also known as niacinamide, is a dietary supplement and precursor of vitamin B3 (Knip et al., 2000). It can inhibit PARP-1 and SIRT-1, as well as *PER1* mRNA expression induced by dexamethasone or trichostatin treatment in NIH-3T3 cells. However, the PARP-1 inhibitor, thymidine, and SIRT-1 inhibitor, sirtinol, do not have the same effect, indicating that nicotinamide-induced repression of *PER1* expression occurs in a PARP-1-dependent or SIRT-1-dependent manner (Xydous et al., 2012). While the full mechanism of *PER1* transcript repression is not yet known, it is clear that this repression can prevent synchronization of peripheral tissues.

Pharmacological manipulation of the clock also occurs under treatment with the mTOR inhibitor, rapamycin. Rapamycin and its analogs, or “rapalogs,” are prescription drugs used to treat a wide variety of conditions, most notably in kidney transplant patients as an immunosuppressant, but also in cancer, diabetes, neurodegenerative diseases, and aging-related pathologies (Li et al., 2014). Metformin, another mTOR inhibitor, is typically used to treat Type 2 Diabetes because it lowers blood sugar levels by activating AMPK. AMPK activation stimulates CK1ε-mediated phosphorylation and subsequent degradation of PER2, lengthening the circadian period (Um et al., 2007). Metformin treatment was shown to cause phase delay in skeletal muscle and phase advance in liver tissue through activation of CKI (Barnea et al., 2012). To our knowledge, there is currently no recommended administration time for rapamycin or metformin. Since mTOR can affect clock function via BMAL1 and CRY1, it is important to coordinate the administration time of these drugs in a way that will not adversely impact the clock. This is especially important as rapamycin and metformin become increasingly popular to treat a wide variety of conditions.

Similarly, drugs that interact with β_2_AR are also capable of affecting clock synchrony. Because β_2_AR activates the p38 pathway, which elevates *BMAL1* and *CLOCK* expression through downstream targets, β_2_AR agonists cause phase shifts up to 2 hours in intestinal tissue, with the direction of the shift dependent on time of administration (Malloy et al., 2012). On the other hand, β-AR antagonists, also called beta blockers, attenuate circadian rhythms of some physiological outputs including heart rate variability, QT interval, and tachyarrhythmic episodes (Munakata et al., 1992; Behrens et al., 1997; Furukawa et al., 2006; Singh and Rabkin, 2021). They also modulate clock timing by suppressing *Bmal1* mRNA expression, as seen in heart tissue of rats (Ushijima et al., 2013). Because of this, it is important to time the administration of beta blockers accordingly. Current evidence supports that taking beta blockers at nighttime is the best chronotherapeutic strategy for lowering nocturnal blood pressure and restoring normal blood pressure rhythms (Portaluppi and Smolensky, 2010). Furthermore, the decrease in nocturnal blood pressure caused by beta blockers taken at bedtime has been shown to decrease nighttime hypertension and improve blood pressure control overall, thus significantly reducing cardiovascular morbidity and mortality (Hermida et al., 2010). Similar research has been performed with calcium channel blockers, which are also used to treat hypertension, but more information is needed to determine the long-term effects of administering calcium channel blockers at bedtime rather than in the early morning (Farah et al., 2013). Future directions should investigate the optimal timing for other drugs targeting receptors in skeletal or cardiac muscle. Evaluating the long-term effects of changing the dosing time should also be a priority.

Some drugs target core clock proteins directly and can be used to treat a variety of conditions. For example, Nobiletin is a polymethoxylated flavone that enhances circadian amplitude of behavioral rhythms in mice by targeting RORs (He et al., 2016). It was shown to attenuate weight gain in mice subjected to diet-induced obesity (He et al., 2016; Nohara et al., 2019). Benzophenone derivatives are another example of clock protein-targeting drugs. They act directly on CRY1 and lengthen the circadian period of U2OS cells in a dose-dependent manner (Kolarski et al., 2021). In like manner, small-molecule agonists of CRY and REV-ERB inhibited glioblastoma stem cell proliferation *in vitro* and tumor growth in mice (Dong et al., 2019). The multilevel oscillatory network of the circadian clock provides ample pharmacological targets that include not only the core clock components such as BMAL1, REV-ERB, and CRY1, but also the ever-growing list of clock modifiers such as AMPK, mTOR, and SIRT1, as discussed here, while the large number of disease states associated with clock disruption supply a multitude of applications.

Notably, chronotherapy is not limited to pharmacological interventions, but should extend to non-pharmacological approaches, especially lifestyle, behavioral, and environmental. For example, bright light therapy and dynamic light therapy have been proposed for treatment of chronically ill patients. Such patients are often unable to go outside during the day and therefore are exposed only to dim indoor lighting during the day and dim light at night, with the latter being relatively bright and capable of disrupting clock functions. This has been associated with delirium, which can prolong hospitalization and increase mortality (Siddiqi et al., 2006). It is thought that delirium develops in these patients because their limited access to bright light attenuates circadian rhythms (Bellapart and Boots, 2012; Fitzgerald et al., 2013; Madrid-Navarro et al., 2015). Fortunately, there is some evidence that light therapy can prevent delirium and reduce post-operative recovery times (Taguchi et al., 2007; Luther and McLeod, 2018; Pustjens et al., 2018). However, there are very few studies that have evaluated the effects of light therapy on hospitalized patients and most of them are limited by small sample sizes. Regardless, light therapy appears to be a promising form of therapy with minimal, if any, adverse effects (Simons et al., 2016). Light therapy has also been used to treat Alzheimer’s disease, Parkinson’s disease, mood disorders, and cancer-related fatigue, highlighting its versatility in treatment applications (van Maanen et al., 2016; Videnovic et al., 2017; Bais et al., 2021; Hung et al., 2021; Kolberg et al., 2021). Future studies should thoroughly investigate the effectiveness of light therapy in a variety of patient groups and settings. They should also identify optimal brightness, duration, and wavelengths of light to be used in such treatments.

There are many other drugs, supplements, and therapeutics that can modulate clock gene expression and rhythms. Here we discussed several most pertinent and recently-developed interventions, but the list is not exhaustive (Chen et al., 2018). However, it is worth mentioning that chronotherapy should be considered when evaluating a treatment regimen as it can significantly affect treatment effectiveness, toxicity, and disease pathogenesis. Finally, sleep, nutrition, and physical activity habits should be considered in conjunction with therapeutics, to coordinate crosstalk with participating signaling pathways.

## CONCLUSION

There are a wide variety of zeitgebers that entrain our internal clocks every day and these include pathophysiological, environmental, lifestyle, and pharmacological cues. They activate a myriad of signaling pathways, many of which are tissue-specific, that reset peripheral clocks. However, some lifestyles disrupt these pathways, such as by delayed sleep and mistimed meals and exercise. These asynchronous zeitgebers lead to internal circadian misalignment, which can have serious health consequences (Megdal et al., 2005; Yousef et al., 2020). Fortunately, small changes in our daily routines, such as reducing short wavelength light exposure at night, restricting food access only during early hours of daytime, avoiding large meals at night, and exercising at more appropriate times than late at night, can have largely positive impacts on life expectancy and quality. More research effort should be put towards identifying genetic markers for different chronotypes and optimizing diet/exercise habits to entrain internal clocks. Chronotherapy is a simple and low-cost, non-pharmacological approach for improving overall health.
